# Enhancement of Tendon Repair Using Tendon-Derived Stem Cells in Small Intestinal Submucosa via M2 Macrophage Polarization

**DOI:** 10.3390/cells11172770

**Published:** 2022-09-05

**Authors:** Xufeng Mao, Liwei Yao, Mei Li, Xiqian Zhang, Bowen Weng, Weilai Zhu, Renhao Ni, Kanan Chen, Linhua Yi, Jiyuan Zhao, Haijiao Mao

**Affiliations:** 1 Department of Orthopaedic Surgery, The Affiliated Hospital of Medical School, Ningbo University, Ningbo 315020, China; 2 Zhejiang Key Laboratory of Pathophysiology, School of Medicine, Ningbo University, Ningbo 315211, China; 3 Ningbo Medical Science Research Institute, Ningbo 315020, China

**Keywords:** Achilles tendon defects, tendon adhesion, treatment, tendon-derived stem cells (TDSCs), small intestinal submucosa (SIS), tendon regeneration

## Abstract

(1) Background: Reconstruction of Achilles tendon defects and prevention of postoperative tendon adhesions were two serious clinical problems. In the treatment of Achilles tendon defects, decellularized matrix materials and mesenchymal stem cells (MSCs) were thought to address both problems. (2) Methods: In vitro, cell adhesion, proliferation, and tenogenic differentiation of tendon-derived stem cells (TDSCs) on small intestinal submucosa (SIS) were evaluated. RAW264.7 was induced by culture medium of TDSCs and TDSCs–SIS scaffold groups. A rat Achilles tendon defect model was used to assess effects on tendon regeneration and antiadhesion in vivo. (3) Results: SIS scaffold facilitated cell adhesion and tenogenic differentiation of TDSCs, while SIS hydrogel coating promoted proliferation of TDSCs. The expression of TGF-β and ARG-1 in the TDSCs-SIS scaffold group were higher than that in the TDSCs group on day 3 and 7. In vivo, the tendon regeneration and antiadhesion capacity of the implanted TDSCs–SIS scaffold was significantly enhanced. The expression of CD163 was significantly highest in the TDSCs–SIS scaffold group; meanwhile, the expression of CD68 decreased more significantly in the TDSCs–SIS scaffold group than the other two groups. (4) Conclusion: This study showed that biologically prepared SIS scaffolds synergistically promote tendon regeneration with TDSCs and achieve antiadhesion through M2 polarization of macrophages.

## 1. Introduction

Achilles tendon (AT) defects are the result of neglected acute Achilles tendon ruptures and the progression of chronic Achilles tendinopathy [1]. Excessive removal of diseased tissue from a tumor during surgery can also lead to AT defects, seriously affecting the function of the ankle joint [2]. Due to its having a lower vascular supply, the injured Achilles tendon has a poor ability to heal itself. Simple sutures are only suitable for patients with small defects without lacerations, while defects larger than 6 cm often require reconstruction with strong mechanically fixed grafts, which has turned out to be a clinical problem that needs to be urgently addressed. Although autologous tendon grafts can resolve some of the small defects, they are more likely to cause structural and functional disturbances at the site where the tendon is taken. 

Postoperative tendon adhesions seriously affect the recovery of limb function and have become another serious clinical problem [3]. With the deepening of the research on tendon adhesion mechanism, scholars have found that, in the first stage of the tendon healing, inflammatory cells, such as macrophages and monocytes precursor, gathered in the injury, a variety of inflammatory cytokines and chemokines in its surrounding are released, the series process allows the recruitment of fibroblasts, promotes collagen deposition, and, eventually, tendon adhesion is inevitable [4,5,6]. These findings suggest that the formation of surface adhesion may be due to the role of macrophages through the cytokine network. It was also found that adhesion formation was negatively correlated with the expression of M2 markers, and the transcription levels of argininase 1 (ARG-1) and mannose receptor 1 (MR-1) on the surface of M2 macrophages in the nonadhesion group were higher [7]. Therefore, reducing the inflammatory response to injured tendons by regulating the polarization of macrophages from M1 to M2 may be effective in preventing the formation of adhesions. 

Various combinations of tissue-engineered scaffolds with mesenchymal stem cells (MSCs) have been used with some success in the treatment of Achilles tendon defects [8,9,10,11] but these combinations do not have early induction of tendon differentiation and antiadhesion properties. In the past few decades, many physical and biofilms made of natural biomaterials or synthetic polymers have been applied to tendon repair and form a physical surrounding around it in an attempt to solve the adhesion problem [12,13,14]. However, subsequent studies have shown that their antiadhesive effect is limited and foreign body reactions are evident [15,16]. MSCs not only provide sufficient cells for tissue reconstruction, but also can achieve the purpose of tissue repair by regulating inflammation [17,18]. Studies have confirmed that MSCs enhance the recruitment of M2 macrophages in tissues by producing immunomodulatory factors [19,20], reducing inflammatory vesicles in macrophages [21], and regulating macrophage polarization. Thus, MSC-derived composite scaffolds are used to reduce foreign body reactions and tendon adhesions [22]. Although the aforementioned scaffolds have had some success in Achilles tendon repair or antiadhesion, few studies have been able to combine the two well.

Taking into account the need for Achilles tendon defect grafts and the mechanisms of adhesion formation, we have developed here the porcine small intestinal submucosa (SIS)/tendon-derived stem cells (TDSCs) scaffold, which possesses characteristics that promote Achilles tendon healing and prevent adhesion. SIS, a naturally occurring collagenous extracellular matrix, has been used to treat tendon defects in animals and has shown enhanced regeneration of tendons [23,24,25]. TDSCs, due to their origin from tendon tissue, not only have the properties of early differentiation into tendon cells, but also have anti-inflammatory properties and are often used as therapeutic seed cells for tendon injury [26,27,28]. Therefore, the combination of the advantages of the two may be the first choice for the development of biocompatible materials that are pro-repair, antiadhesion, and biodegradable. SIS provides a large amount of collagenous tissue for Achilles tendon regeneration and a suitable cellular environment for TDSCs, which both induces early tendon differentiation and promotes macrophage polarization, inhibits inflammation, and ultimately promotes Achilles tendon repair. In this study, we attempted to develop an SIS scaffold for Achilles tendon surgery with TDSCs. Biocompatibility and bioactivity of SIS seeded with TDSCs were evaluated in vitro, and potential for Achilles tendon repair was evaluated in vivo. The effects of SIS/TDSCs scaffolds on macrophage polarization in vitro and in vivo were investigated. 

## 2. Materials and Methods

### 2.1. Ethics Statement

We followed the institution’s guidelines in regards to the feeding, care, operation, and treatment of experimental animals. The Animal Research Ethics Committee of Ningbo University in China had approved all experiments (NBU20220114). 

### 2.2. Material Preparation

#### 2.2.1. SIS Scaffold

Cook Biotech Inc. kindly provided lyophilized SIS scaffolds. The scaffolds were drilled into 5-mm-diameter wafers using a hole punch (20H70, Miltex, Loznica, Serbia) in vitro, and 1.0 cm × 1.5 cm in vivo.

#### 2.2.2. SIS Hydrogel Coating

Fresh pig small intestine was mechanically treated and only the submucosa was retained, and SIS was soaked using a 1:1 volume ratio of methanol (#10014118, Sinopharm Group, Shanghai, China) and chloroform (#20210406, Sinopharm Group, China) for 12 h at a time, then washed with sterile phosphate-buffered saline (PBS) (P1010, Darthill Biotech, Shanghai, China) solution for 30 min, and the above steps were repeated 2 times. SIS was treated with 0.05% trypsin (T8150, Solarbio, Beijing, China) solution for 12 h, washed again with PBS once, and the above steps were repeated 3 times. Immediately afterwards, the SIS was immersed in a 0.5% SDS (S8010, Solarbio, Beijing, China) solution for 4 h, then washed with PBS for 30 min, and the above steps were repeated 2 times. Finally, it was soaked in 75% alcohol (#20220106, Halberd, Ningbo, China) for 20 min and freeze-dried at −80 °C for 2 h. After freeze-drying, the tissue was cut into powder form and digested with 1 mg/mL pepsin (P8160, Solarbio, Beijing, China), pH was maintained in the range of 1–2 with 0.04 mol/L hydrochloric acid (#21080604, Sinopharm Group, Shanghai, China), and it was stirred with magnetic beads to finally obtain SIS hydrogel. The hydrogel of 10 mg/mL was incubated in a 96-well plate overnight, and then discarded and dried for later use.

### 2.3. Assessments of Biocompatibility

#### 2.3.1. Isolation and Culture of TDSCs 

Eight-week-old Sprague–Dawley rats weighing 200–300 g were used. Isolation and culture of TDSCs from rats were carried out as described previously [26,29,30]. Briefly, both Achilles tendons of the rats were carefully severed without taking out any muscles and tissues were removed from the surrounding areas. After incubation with sterile PBS and three 5-min rinses, they were trimmed into 1 mm × 1 mm × 1 mm tissue blocks with sterile tissue shears. A syringe was used to transfer the tissue suspension into a centrifuge tube, which was centrifuged at 300× *g* for 5 min. Then, the excess PBS was sucked up and the tissue blocks were digested for 2.5–3 h at 37 °C with 3 mg/mL type I collagenase (C8140, Solarbio, Beijing, China). At last, a single-cell suspension was yielded through a 70-μm cell strainer (#352350, Corning Falcon, Thermofisher Scientific, Waltham, MA, USA), which resuspended in Dulbecco’s modified Eagle medium (2192823, Gibco, Shangai, China) with 10% fetal bovine serum (#2128194, XP Biomed Ltd., Shanghai, China), penicillin–streptomycin (#15140163, TRANSGEN, Beijing, China). Cells were cultured at 37 °C in 5% CO_2_ for 2 days until adherent cells were detected. The complete medium was then replaced. At day 5–7, the passage 0 cells were mixed together, while the cell density converged to 80%. The passages 3 cells were used for all experiments.

#### 2.3.2. Flow Cytometry Assay of TDSCs 

Flow cytometry assay of TDSCs was carried out as described previously [30,31]. TDSCs (1 × 10^6^) were incubated with 1 µg of AF647-, PE-, PE-Cy7-, or FITC-conjugated specific mouse to rat monoclonal antibodies for 20 min at 4 °C. PE- or FITC-conjugated isotype-matched IgGs (#65209, Abclonal, Wuhan, China) were used as controls. After rinsing with PBS at 300 g for 5 min, the stained cells were resuspended in 400 μL of PBS with 10% FBS (#2128194, XP Biomed Ltd., Shanghai,, China) and analyzed by flow cytometry (Beckman Cytoflex, Beckman Coulter, Brea, CA, USA). Approximately 10^5^ events were counted per sample. The FACSCAN program (Beckman Cytoflex) was used to calculate the percentage of positive signaling cells. The antibodies, including anti-CD29 (#562153, Becton Dickinson, Franklin Lakes, NJ, USA), anti-CD44 (#550974, Becton Dickinson, Franklin Lakes, NJ, USA), anti-CD90 (#551401, Becton Dickinson, Franklin Lakes, NJ, USA), anti-CD34 (sc-7324, Santa Cruz Biotechology, Dallas, TX, USA), and anti-CD31 (FITC-65058, Proteintech, Wuhan, China), were used in this study.

#### 2.3.3. Trilineage Differentiation of TDSCs 

Trilineage differentiation of TDSCs was carried out as described previously [30]. The adipogenic, osteogenic, and chondrogenic differentiation kits were used to examine the trilineage differentiation ability of TDSCs (TSGU-D102R, TSGU-D101R, TSGU-D203, Hai xing Biosciences, Shenzhen, China). Briefly, TDSCs were seeded into a 48-well plate with a density of 5 × 10^4^ cells/well; then, adipogenic, osteogenic, and chondrogenic induction media were administered separately. After 10–14 days of incubation, they were fixed with 4% paraformaldehyde (P8430, Sinopharm Group, Shanghai, China) for 20 min and then were stained with Oil red O, Alizarin red, and Alcian blue using the above kits (#211220C02, 220120S01, 210928C02, Hai xing Biosciences, Shenzhen, China).

#### 2.3.4. Colony Formation Assay

Colony formation assays were used to assess colony-forming capacities. Briefly, 200 cells/well of TDSCs were seeded into a 6-well plate, and they were cultured for 10 days in complete medium. Afterward, formed colonies were fixed with 4% paraformaldehyde (P8430, Sinopharm Group, Shanghai, China) for 20 min and stained with 1% crystal violet (C10665923, Solarbio, Beijing, China) for 15 min. The stained colonies were then photographed.

#### 2.3.5. Proliferation, Adhesion, and Differentiation of Tendon Stem Cells on SIS

A total of 2000 cells were sequentially inoculated on SIS hydrogel coating in 96-well plates, and those without coating served as the control group. The experiment of proliferation was assessed by CCK8 (#O21101, TRANSGEN, Beijing, China); 10 µL CCK8 reagent was added into each well for 2 h incubation. The absorbance (450 nm) of each well was determined to evaluate the cell viability per standard protocol outlined by the manufacturer’s instruction.

Adhesion assay was applied to evaluate cell adhesion ability. A total of 1 × 10^3^ cells/mL were seeded onto SIS scaffold. After incubation at 37 °C for 1, 3, 5, and 7 days, the cells were fixed with 4% paraformaldehyde (P8430, Sinopharm Group, Shanghai, China) for 20 min, stained with anti-rabbit COL1A1 (A16891, Abclonal, Wuhan, China) and DAPI (C0065, Solarbio, Beijing, China), and then counted in five randomly selected microscopic fields.

The sterilized SIS scaffolds were soaked in complete culture medium for 48 h before seeding. The cells were planted into a 96-well culture plate with the concentration of 5 × 10^4^ cells/mL. The next day, the SIS scaffolds containing the cells were transferred to new holes for further culture. The holes with SIS scaffold were our experimental group, while those without were the control group. The levels of SCX, TNMD, COL1A1, and COL3A1 were measured by qRT-PCR at days 7 and 14. β-Actin was the housekeeping gene.

### 2.4. M1 and M2 Polarization Model

LPS (L8880, Solarbio, Beijing, China) combined with IFN-γ (P00106, Solarbio, Beijing, China) or IL-4 (HZ-1004, Proteintech, Wuhan, China) was used to construct the model of M1 and M2 polarization in RAW264.7 cells [32,33,34]. Briefly, RAW264.7 cells were inoculated in 24-well plates at a density of 50 × 10^4^ per well. In the control group, no treatment was labeled as M0 macrophages. In the model of the M1 polarization group, LPS 100 ng/mL combined with IFN-γ 20 ng/mL was given, while IL-4 20 ng/mL was given in the M2 polarization group. RNA from the cells were extracted after 12 h treatment, and then they were converted into cDNA. The levels of TGF-β, ARG-1, TNF-α, and iNOS of RAW264.7 were assessed using qRT-PCR.β-Actin as the housekeeping gene.

### 2.5. Induction of RAW264.7 Cells by Culture Media

The culture media of TDSCs and TDSCs–SIS scaffold were collected and centrifuged for 500× *g*/5 min. They were prepared to induce RAW264.7 cells. Briefly, RAW264.7 cells were inoculated in 24-well plates at a density of 50 × 10^4^ per well. In the control group, cells received no treatment. In the induced group, cells received the culture media of TDSCs or TDSCs–SIS scaffold, with a ratio of 7 to 2 of complete medium to supernatant. The levels of TGF-β, ARG-1, TNF-α, and iNOS of RAW264.7 cells were measured by qRT-PCR. β-Actin was the housekeeping gene. Our study was focused on days 3, 7, and 10.

### 2.6. qRT-PCR

The gene expression of SCX, TNMD, COL1A1, and COL3A1 was performed after coculture for 7 and 14 days by quantitative reverse-transcription PCR (qRT-PCR) (*n* = 3). The gene expression of TGF-β, ARG-1, TNF-α, and iNOS was performed in the same way after coculture for 3, 7, and 10 days. We extracted the total RNA from the cells using TRIzol reagent according to the manufacturer’s protocol (P40927, Takara, Dalian, China). Reverse transcription of full-length cDNA was then performed using a cDNA syntheses kit (AT311, TRANSGEN, Beijing, China). The qRT-PCR was conducted according to the manufacturer’s instructions (P41014, TRANSGEN, Beijing, China). β-Actin was the housekeeping gene. PCR primer sequences are shown in Table 1.

### 2.7. Immunofluorescence

Protein expression of COL1A1 in each group was detected with confocal microscopy (LEICA TCS SP8) after coculture for 1 and 7 days. The samples were fixed with 4% paraformaldehyde, sealed with 10% goat serum (B900780, Proteintech, Wuhan, China), and then incubated overnight with primary antibody (COL1A1, A16891, ABclonal, Wuhan, China) 1:100. After rewarming at room temperature, the samples were incubated with secondary anti-rabbit IgG (AS039, ABclonal, Wuhan, China) 1:200 for 1 h. Subsequently, nuclei were stained using DAPI. Each sample randomly selected 3 vision, and each group of 3 samples, a total of 2 time nodes, measured 18 vision. 

### 2.8. Assessments of In Vivo Repair

#### 2.8.1. Rats and Experimental Design

A total of 60 male Sprague–Dawley rats were purchased from the Laboratory Animal Center of Ningbo University of China. Twelve of these rats were 8 weeks old and were used for the preparation of TDSCs. The remaining 48 rats, weighing 200 g to 250 g, were used for animal experiments.

The 48 rats were randomly assigned to 4 groups: (1) TDSCs–SIS scaffold group (TDSCs–SIS) containing TDSCs (10^6^)-loaded SIS scaffold; (2) SIS scaffold control group (SIS) containing SIS scaffold, without loaded TDSCs; (3) blank defect group (Defect) without containing SIS scaffold and TDSCs, which were unrepaired defect sites; and (4) normal group (Normal), which were not suffering any injury. In total, *n* = 8, half for histological analysis and half for biomechanical testing. All animals were sacrificed at 1, 2, and 3 months post-surgery. 

#### 2.8.2. Surgical Protocol

All animal experiments were carried out in the Animal Experiment Center of Ningbo University. The rats were anesthetized with 1% pentobarbital sodium intraperitoneally, and the Achilles tendons were exposed and the flexor digitorum tendons were removed. A 3-MM-diameter defect was created by a perforator (DL1919, Deli, Ningbo, China) when the Achilles tendon was stretched by an assistant. The corresponding experimental materials were taken out, the defect holes were wrapped, both ends were fixed with 4-0 tendon line (QGBDBL, JNJ, Shanghai, China ), the skin was sutured layer by layer, sterile auxiliary materials were bandaged, and plaster fixation was performed. The rats were placed in lateral supine position on a thermostatic resuscitation table, monitored by a special person until they woke up, and then transferred to a cage box. The rats were evaluated daily for signs of disease and for infection of surgical wounds. After 1, 2, and 3 months, rats were sacrificed by intraperitoneal injection of excess sodium pentobarbital. The Achilles tendon was taken from all specimens and the surrounding tissue removed. 

#### 2.8.3. Macrographic Examination

Adhesion was graded in accordance with the following system [24]: in grade 0, there is no adhesion; in grade 1, the vascularity is opaque, transparent, and filmy; in grade 2, the vascularity is opaque, translucent, and filmy; and, in grade 3, the vascularity is opaque and larger vessels are present. Two blinded surgeons observed independent scope adhesion formation while animals were sacrificed. Anesthesia was applied to rats at 1, 2, and 3 months following the operation. Both surgical sites in the legs were exposed in order to observe whether infection or abnormal secretion occurred. 

#### 2.8.4. Tissue Sample Preparation

Samples of Achilles tendon were taken after the first month, the second month, and the third month following postoperative surgery. Each sample was paraffin-embedded and sectioned into 5-μm-thick sections. PBS was immersed in samples for biomechanical tests, and the test was completed within four hours (*n* = 4 for each group).

#### 2.8.5. Histopathological Analysis

Hematoxylin and eosin (H&E) staining, Masson staining, and immunohistochemical staining were performed on the collected specimens. The specimens were fixed with 4% paraformaldehyde overnight, dehydrated, transparent, immersed in wax, embedded in paraffin, and then coronal sectioned (5 μm) along the longitudinal axis of the tendon. H&E staining and Masson staining were performed according to standard protocols (G1121, G1345, Solarbio, Beijing, China). The immunohistochemical staining was conducted using the DAB (ab161117, abcam, Shanghai, China) staining method. Briefly, after dewaxing and rehydration, the slides were repaired by enzyme repair method for antigen repair. After neutralization by 0.08% trypsin (T8150, Solarbio, Beijing, China), the tissues were covered and incubated at 37 °C for 2 h. After sealing with hydrogen peroxide (10011218, Sinopharm Group, Shanghai, China) and 10% goat serum, the slides were incubated with anti-SCXA (ab58655, abcam, Shanghai, China) 1:100, anti-Tenomodulin (ab203673, abcam, Shanghai, China) 1:100, anti-rabbit COL1A1 (A16891, ABclonal, Wuhan, China) 1:100, anti-rabbit COL3A1 (A3795, ABclonal, Wuhan, China) 1:100, anti-rabbit CD68 (25747-1-AP, Proteintech, Wuhan, USA) 1:100, and anti-rabbit CD163 (16646-1-AP, Proteintech, Wuhan, USA) 1:100 overnight at 4 °C. Subsequently, secondary antibody (CR2107161, Servicebio, Wuhan, China) 1:200 and DAB (CR2103183, Servicebio, Wuhan, China) chromogenic agents were added sequentially according to the manufacturer’s instructions. Finally, the nucleus was stained with hematoxylin. We used the established scoring system for histology to analyze how the scores on HE-stained slides changed after treatment [35]. The intact group scored 20 points. A scoring system is presented in Table S1.

#### 2.8.6. Biomechanical Testing

The biomechanical parameters were assessed through a biomechanical testing machine (#3366, INSTRON, Shanghai, China). During the first, second, and third month following the surgery, specimens of the Achilles tendon, along with bony structures and muscle tips, were completely removed. One leg of each rat was randomly selected for the tensile test. The specimens were measured by caliper for their diameter and length. Both ends of the specimens were fastened to the upper and lower splints of the testing machine. The tendon was pulled at a constant speed of 10 mm/min. The maximum loading and ultimate stress were calculated. 

### 2.9. Statistical Analysis

Data were presented as the mean ± SD. We used the Student’s t-test to examine the differences between the two groups. One-way analysis of variance (ANOVA) was used to compare two or more groups; then, Bonferroni post hoc multiple comparisons were performed. The statistical analysis was performed using SPSS 19.0 (SPSS, Inc., Chicago, IL, USA). Statistical tests are described in the figure legends. Bar graphs and dot plots were generated with GraphPad Prism 9 and show the mean ± S.D.

## 3. Results

### 3.1. Characterization of TDSCs

**Figure 1:**
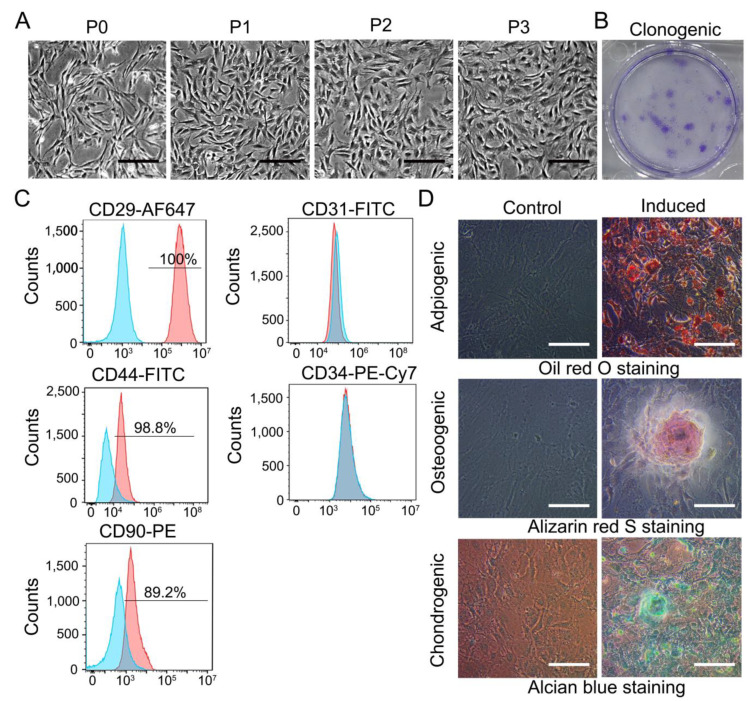
Characterization of TDSCs. (**A**) The cells presented spindly like. (Bar: 200 μm.) (**B**) Clonogenesis of TDSCs, using crystal violet staining. (**C**) TDSCs were positive for CD29, CD44, and CD90 but negative for CD31 and CD34. TDSCs, tendon-derived stem cells. (**D**) Adipogenesis, osteogenesis, cartilage-like, using Oil red O, Alizarin red S, and Alcian blue staining, respectively. Bar: 100 μm.

As described in Materials and Methods, TDSCs were isolated by a protocol. P0-P3 generation of TDSCs were presented in (Figure 1A). P0 cells exhibited clonal proliferation of TDSCs, which passed from generation to generation every 2–3 days. Finally, uniform long spindle cell morphology was formed in P3. TDSCs also had some ability to form clones (Figure 1B). The expression of TDSC surface markers on the cells were examined using flow cytometry to confirm that the tendon-derived cells were stem cells. Results demonstrated the presence of the markers CD29 (100%), CD44 (98.8%), and CD90 (89.2%) but were negative for the endothelial and hematopoietic markers CD31 and CD34 (Figure 1C). It was shown in an in vitro induction model that TDSCs could be successfully differentiated into lipogenic, osteogenic, or chondrogenic cell lines (Figure 1D). 

### 3.2. SIS Scaffold Facilitated Cell Adhesion and Tenogenic Differentiation of TDSCs, While SIS Hydrogel Coating Promoted Proliferation of TDSCs 

**Figure 2:**
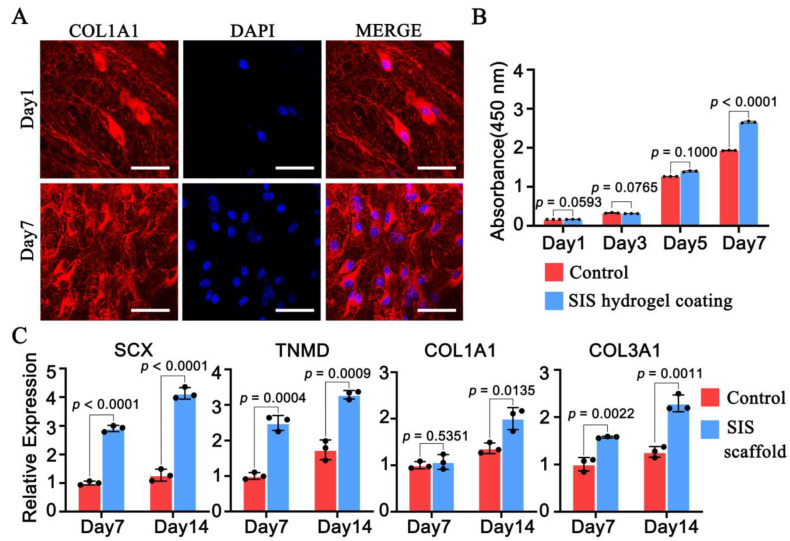
Small intestinal submucosa (SIS) scaffold facilitated cell adhesion and tenogenic differentiation of tendon-derived stem cells (TDSCs), while SIS hydrogel coating promoted proliferation of TDSCs. (**A**) TDSCs had obvious adhesion effect on SIS scaffold, which was observed by COL1A1 and DAPI of IF staining on day 1 and day 7. COL1A1, pro-alpha1 chains of type I collagen. DAPI, 4′,6-diamidino-2-phenylindole. IF, immunofluorescence. Bar: 50 μm. (**B**) SIS hydrogel coating promoted TDSC proliferation through CCK8 proliferation assay on day 7. (**C**) Total RNA was extracted on days 7 and 14 and the expression of genes related to tenogenic differentiation were detected by quantitative real-time qPCR. Data in (**B**,**C**) are mean ± S.D. (*n* = 3) biological replicates from one representative experiment analyzed with Student’s *t*-test for significance.

To verify the presence of TDSCs on SIS, we evaluated the situation using type I collagen fluorescence staining. Both SIS scaffold and TDSCs were stained by type I collagen immunofluorescence staining (Figure 2A), from a small amount of cell adhesion on day 1 to every field on day 7. The SIS hydrogel coating facilitated the proliferation of TDSCs, with the experimental group outperforming the control group on day 7 (Figure 2B). Gene expression profiles of TDSCs seeded on SIS scaffold at 7 days and 14 days were analyzed by qRT-PCR (Figure 2C). The expression levels of SCX, TNMD, COL1A1, and COL3A1 in the SIS group all upregulated compared with the control group at 7 days and 14 days, except for COL1A1 on day 7.

### 3.3. Transplantation of TDSCs–SIS Scaffold to Repair Achilles Tendon Defect in Rats

**Figure 3:**
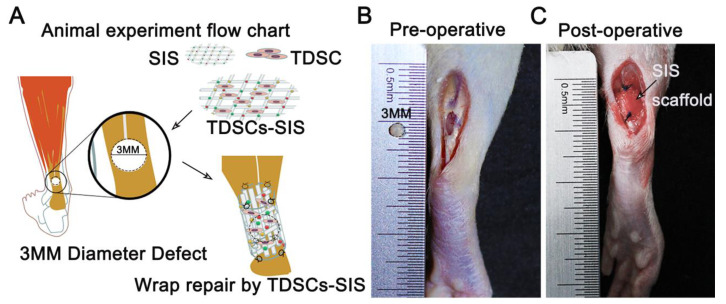
An animal model of repairing Achilles tendon defects with TDSCs–SIS scaffolds. (**A**) The Achilles tendon defects repaired by TDSCs–SIS scaffolds of animal experiment flow chart. (**B**) A special hole punch was used for modeling Achilles tendon defects with a diameter of 3 mm. (**C**) Immediate postoperative image of the repair of Achilles tendon defect with TDSCs–SIS scaffolds.

To assess the therapeutic effects of the TDSCs–SIS scaffold, we established bilateral Achilles tendon defects of rats and then transplanted TDSCs–SIS scaffold/SIS scaffold into the injured site using 4-0 micro tendon lines (Figure 3A–C).

### 3.4. TDSCs–SIS Scaffold Improved Tendon Healing through Gross and Histological Evaluation

There were no complications associated with anesthesia or surgery in any of the animals and no fevers or abnormal appetites in the animals. The animals moved normally, with the exception of some joint movement while immobilized in the cast. After removal of the casts, these animals regained full use of the surgical legs over a period of 4 to 6 weeks. Despite varying amounts of weight bearing on both legs, all the animals were able to walk normally after seven weeks with no lameness.

**Figure 4:**
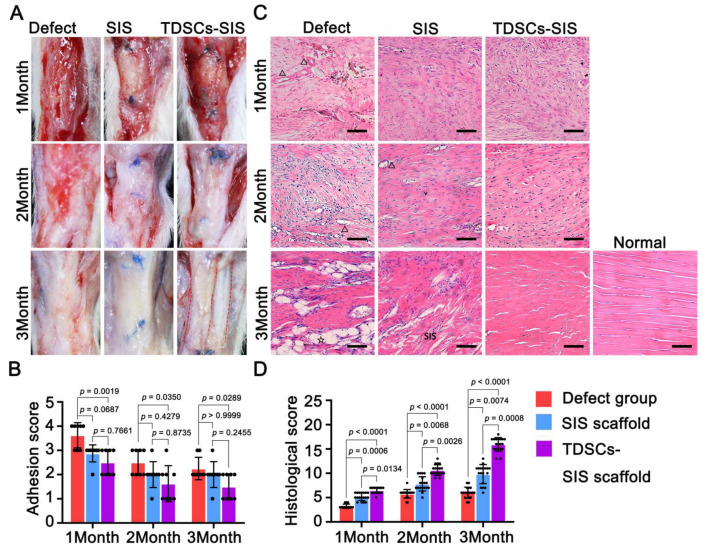
TDSCs–SIS scaffold (TDSCs–SIS) could have antiadhesion and promote histological healing. (**A**) At the third month, the SIS scaffold in the TDSCs–SIS group was degraded earlier than all groups, exposing 2 strands of Achilles tendon tissue, and no peritendinous adhesions occurred by macrographic evaluation, *n* = 8. (**B**) The adhesion score through macrographic evaluation, *n* = 8. (**C**) Histological sections were stained with hematoxylin and eosin in the Achilles tendon defects of the blank defect or implantation with scaffold (SIS and TDSCs–SIS) after 1, 2, and 3 months, *n* = 4. Bar: 75 μm. △ represents vessel. ☆ represents adipocyte. (**D**) The histological score; 20 points is a normal Achilles tendon, *n* = 4. Data in (**B**) (*n* = 8) and (**D**) (*n* = 4) are mean ± S.D. analyzed by one-way analysis of variance (ANOVA) with Bonferroni post hoc multiple comparisons.

The degree of adhesion in the regenerated Achilles tendon with SIS scaffold involvement was better than in the defect group and was most pronounced at the third month postoperatively (Figure 4A). The regenerated Achilles tendon group with TDSC involved in the repair showed partial degradation of the SIS scaffold by the second postoperative month and complete degradation by the third postoperative month, while no degradation was detected in the SIS scaffold group. From the second month on, the periphery of the Achilles tendon in the TDSCs–SIS scaffold group was smooth, with light adhesion, and thinner than the other two groups, which were consistent with adhesion scores (Figure 4B). Furthermore, the regenerated Achilles tendon was lined up in two bundles in the third postoperative month, a continuation of the gastrocnemius and flounder muscles, although it did not eventually form a bundle like a normal Achilles tendon (Figure 4A).

H&E (Figure 4C) and Masson’s trichrome (Supplementary Figure S1) staining revealed that tendon in the TDSCs–SIS scaffold treatment group at the third month showed more continuous and regular arrangement in contrast to disorganized tendon in the SIS scaffold control group and blank defect group. 

After 1 month of treatment, the histology of the Achilles tendon tissue was slightly different, although the histological scores were all lower. The TDSCs–SIS scaffold treatment group had more filling and less voids in the defect area, accompanied by a small number of small vascular infiltration, while the blank defect group had less filling and more voids, accompanied by vessels of different sizes, and the SIS scaffold group was located in the middle. However, SIS scaffold did not appear in the defect area. 

After 2 months of treatment, SIS scaffold began to appear in the defect area of the TDSCs–SIS scaffold group, without blood vessels. However, no SIS scaffold was found in the SIS scaffold group, but the tissue in the area of defect became dense with small vessels. The gap in the blank defect group was reduced compared to itself 1 month before, with small vessels, no inflammatory cells, and no large vessels.

After 3 months of treatment, there were significant differences in histology of Achilles tendon tissue. The TDSCs–SIS scaffold group presented a wavy shape with no SIS scaffold residue, which was similar to the normal Achilles tendon tissue. A large number of SIS scaffolds were filled with defect area in the SIS scaffold group. Meanwhile, the blank defect group was full of fat tissue and blood vessels. Histological scores also confirmed these results (Figure 4D). 

### 3.5. TDSCs–SIS Scaffold Contributes to Biomechanical Property Recovery

**Figure 5:**
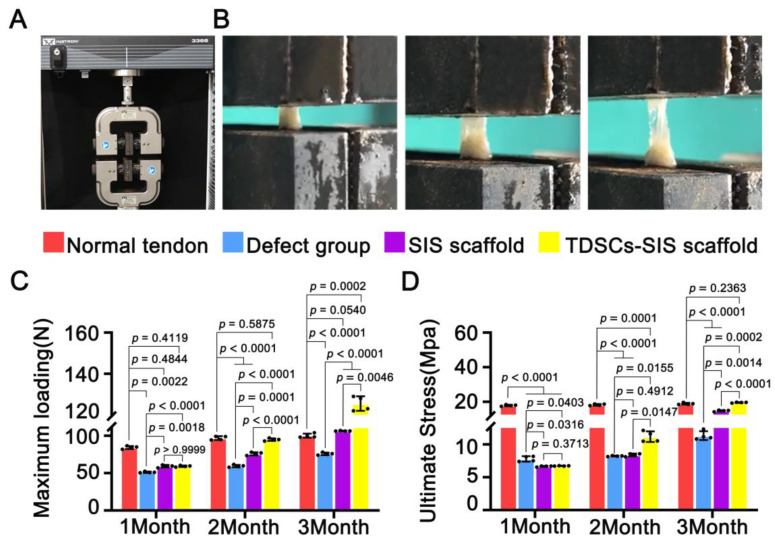
TDSCs–SIS scaffold facilitated biomechanical recovery of the Achilles tendon. (**A**,**B**) Biomechanical test was performed; the Achilles tendon was stretched at a uniform rate until it was completely stretched. (**C**,**D**). Biomechanical properties, including maximum loading and ultimate stress, were measured; the maximum loading and ultimate stress in TDSCs–SIS scaffold group were better than those in the SIS scaffold and defect groups from the second month onwards and this trend was amplified in the third month. *n* = 4. Data in (**C**,**D**) are mean ± S.D. (*n* = 4), analyzed by ANOVA with Bonferroni post hoc multiple comparisons.

Mechanical results shown that the biomechanical parameters of the regenerated Achilles tendon were improved with the extension of time after repair with TDSCs and SIS scaffold, especially with the combination of the two. The process of Achilles tendon stretching is shown in (Figure 5A,B). The maximum loading was higher in both the TDSCs–SIS scaffold group and the SIS scaffold group at month 1 postoperatively than in the defect group, but the difference was not statistically significant between them until month 2. Compared to the defect and SIS group, statistical differences in ultimate stress began to appear in the TDSCs–SIS scaffold group at month 2 and, although this difference was not yet as significant, it also performed better than month 1. At month 3, the maximum loading and ultimate stress in the TDSCs-SIS scaffold group were significantly higher than in other groups, and there was also significant difference between the SIS scaffold group and the blank defect group (Figure 5C,D). Furthermore, at month 3, the ultimate stress reached the normal level, while the maximum loading was above normal. All samples failed at the place of regenerated Achilles tendon and all samples were tested at the same condition and parameter.

### 3.6. TDSCs–SIS Scaffold Promoted Regenerated Achilles Tendon and Regulated Extracellular Matrix Formation

**Figure 6:**
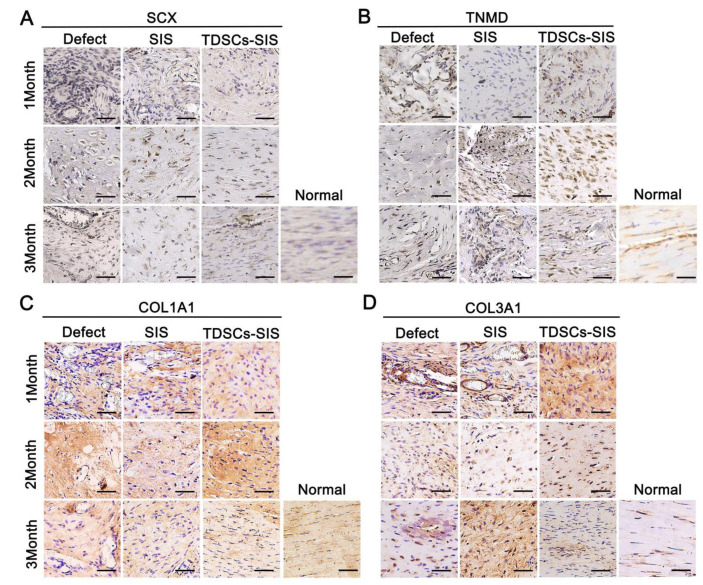
TDSCs–SIS scaffold facilitated Achilles tendon regeneration. (**A**,**B**) Expression of SCX and TNMD was evaluated at 1, 2, and 3 months by immunohistochemistry assay. (**C**,**D**) Expression of the extracellular-matrix-related factors COL1A1 and COL3A1 were evaluated after TDSCs–SIS scaffold treatment at 1, 2, and 3 months. Bar: 50 μm.

We studied the effect of TDSCs–SIS scaffold on regenerated Achilles tendon, TNMD, SCX, and tendon-matrix-related factors, COL1A1 and COL3A1. The expression of SCX and TNMD in the TDSCs–SIS scaffold group was significantly higher than that of other groups at month 2 and 3 after operation (Figure 6A,B). At month 2, the expression of type I collagen in TDSCs–SIS scaffold group was higher than that in other groups, and then returned to normal at month 3 (Figure 6C). The expression of type III collagen in the TDSCs–SIS scaffold group was the highest at month 1, while that in the SIS scaffold group appeared in month 3 after surgery. In addition, type III collagen expression was difficult to find in the TDSCs–SIS scaffold group in month 3 (Figure 6D).

### 3.7. Effect of TDSCs and SIS Scaffold on Macrophage Polarization toward the M2 Phenotype

**Figure 7:**
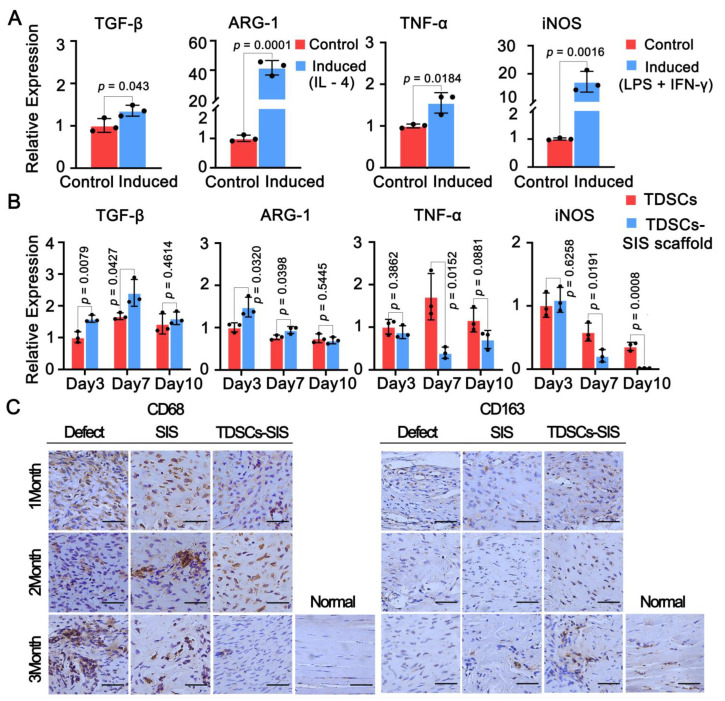
TDSCs–SIS scaffold facilitated macrophage polarization toward the M2 phenotype. (**A**) LPS and IFN-γ or IL-4 were used to construct the model of M1 and M2 polarization in RAW264.7 cells. (**B**) The expression effect of TGF-β and ARG-1 in TDSCS–SIS scaffold group was better than that in TDSCs group on day 3 and 7, when RAW264.7 was induced by culture medium of both groups. (**C**) The M1-like phenotype, CD68, was highly expressed in the defect group and lowly expressed in the SIS scaffold group and TDSCs–SIS scaffold group. The M2-like phenotype, CD163, was low in the defect group and high in the TDSCs–SIS scaffold group. Bar: 75 μm. Data in (**A**,**B**) are mean ± S.D. (*n* = 3) biological replicates from one representative experiment analyzed with Student’s *t*-test for significance.

Considering the ability of TDSCs and SIS scaffold to regulate macrophage polarization, the expression of M1 and M2 was detected in vitro and in vivo, respectively. In vitro, M1 polarization model successfully expressed TGF-β and ARG-1, while the TNF-α and iNOS were also detected in the M2 polarization model (Figure 7A). Then, we used culture medium of TDSCs and TDSCs–SIS scaffold to stimulate RAW264.7, and the results showed that the relative expressions of TGF-β and ARG-1 in the TDSCs–SIS scaffold group were better than those in the TDSCs group on the 3rd and 7th day after stimulation, while this trend was not statistically significant on the 10th day (Figure 7B). Correspondingly, the expression of proinflammatory genes TNF-α and iNOS in TDSCs–SIS scaffold group was significantly lower than that in the TDSC group on the 7th day. In vivo, immunohistochemical staining showed the expression of CD68 in the defect group was higher than that in the other two groups, while the expression of CD68 in the SIS group and TDSCs–SIS scaffold group showed a downward trend, especially in the TDSCs–SIS scaffold group (Figure 7C). In contrast, CD163 expression was difficult to detect in the defect group, and was not detected until month 3 after surgery in the SIS scaffold group, while it began to appear at month 3 after surgery in the TDSCs–SIS scaffold group (Figure 7C).

**Figure 8:**
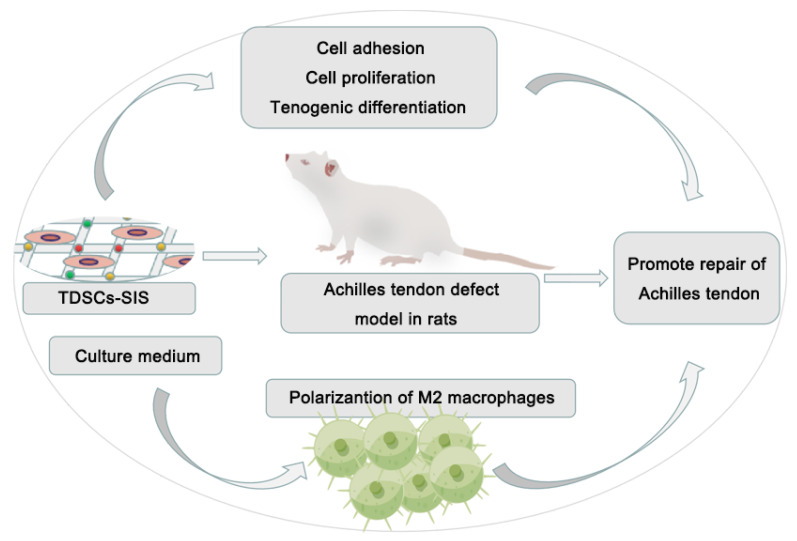
Summary illustrations for TDSCs–SIS in in vitro and in vivo assessments. In vitro SIS promoted the adhesion, proliferation, and tenogenic differentiation of TDSCs, while the culture media of TDSCs–SIS promoted the polarization of M2 macrophages. In vivo TDSCs–SIS scaffold promoted the regeneration of Achilles tendon. The synergy of in vitro and in vivo work together to promote repair of the Achilles tendon.

A summary illustration is shown in Figure 8. In vitro SIS promoted the adhesion, proliferation, and tenogenic differentiation of TDSCs, while the culture media of TDSCs–SIS promoted the polarization of M2 macrophages. In vivo TDSCs–SIS scaffold promoted the regeneration of Achilles tendon. The synergy of in vitro and in vivo work together to promote repair of the Achilles tendon.

## 4. Discussion

The most important finding of this study is the application of the TDSCs–SIS scaffold to the Achilles tendon defect model; after the wrapped repair of the Achilles tendon defect area, it not only reconstructed and repaired the Achilles tendon defect, but also isolated the adhesion between the surrounding soft tissues and the Achilles tendon defect area, while delivering its loaded TDSCs to the defect area precisely, providing a good cellular microenvironment for the proliferation and differentiation of TDSCs, and, also, it can regulate the inflammatory response during Achilles tendon healing more accurately.

TDSCs play the vital role for tendon injury healing with its excellent tenogenic differentiation, including formation of tenocytes and extracellular matrix [36,37,38]. The induced differentiation experiment in the present study showed that TNMD, SCX, COL1A1, and COL3A1 were highly expressed in the induced group. Repairing Achilles tendon defects requires materials with a good mechanical strength and biocompatibility. Compared with synthetic materials, natural biomaterials exhibit excellent biomimicry properties and a natural composition [39,40,41]. SIS was previously described as a naturally occurring, cell-free, collagenous extracellular matrix (ECM) that contains a range of bioactive molecules [42]. The superior biocompatibility and angiogenic properties of SIS have made it widely used in tissue repair, including tendon tissue engineering. Thore Zantop et al. used SIS to repair a 2 mm defective Achilles tendon, demonstrating the ability to recruit a population of bone-marrow-derived cells to participate in the tendon remodeling process when SIS is used as a scaffold for repair [43]. Thomas W. Gilbert et al. showed similar results when applying SIS to canine Achilles tendon repair [25]. Zhicheng Song et al. used three-layer SIS composite tendon cells to repair abdominal wall defects in rats, demonstrating that tendon cells and SIS can generate engineered tendon membranes in vivo with better mechanical loading and biocompatibility [44].

In the present study, we seeded TDSCs on SIS hydrogel coating to promote proliferation and on SIS scaffolds to enhance their adhesion and differentiation. The environment provided by the SIS hydrogel coating proved to be more conducive to the proliferation of TDSCs. In the absence of induction reagents, SIS scaffold was able to differentiate TDSCs into a tendon-forming cell phenotype as early as 7 days. SIS scaffold proved to be very compatible with tendon stem cells in vitro. The cell scaffold structure can, therefore, be implanted directly into the body without the need for prolonged in vitro culture for tendonogenic differentiation. In vivo, studies further demonstrated that SIS scaffold and TDSCs synergistically promoted Achilles tendon regeneration in a 12-week rat Achilles tendon defect model, with significant infiltration of SIS scaffold fibers into the newly formed Achilles tendon, indicating that SIS collagen fibers were reused during Achilles tendon regeneration, further demonstrating the good biocompatibility of SIS in Achilles tendon tissue engineering. The process may be triggered by increased cell recruitment, growth, and differentiation. The mechanism is based on the similarity of the tissue composition between SIS and TDSCs, thereby providing the TDSCs with a good microenvironment and sending multiple repair signals which are likely to be emitted by the entire SIS microenvironment [45]. In proliferation or damage, Col3 increases in amount. During the maturation stage of tendon remodeling, Col1 is found in the mature matrix of the tendon. A structural change occurs in the tendon when collagen fibers organize along the longitudinal axis, restoring stiffness and tensile strength by replacing Col3 with Col1 [46,47]. In our experiment, the expression of COL3A1 was the highest in the TDSCs–SIS scaffold group at the first month, which may be due to the synergistic effect in SIS and TDSCs–SIS scaffold groups to better secrete COL3A1. Meanwhile, COL1A1 also showed the highest expression in the TDSCs–SIS group at the second month after surgery. 

Macrophage polarization is a process thought to play a pivotal role in tissue reconstruction after injury [48]. At the site of tendon-to-bone repair, injection of fresh bone marrow can expand the tendon-to-bone contact surface and promote tendon-to-bone healing. M2 macrophage polarization is involved in regulating the inflammatory process [49]. An electrospun polycaprolactone/silk fibroin (PCL/SF) composite fibrous scaffold functionalized with mesenchymal stem cell (MSC)-derived extracellular matrix (ECM) can effectively inhibit inflammation and prevent tendon adhesions by promoting the polarization of M2 macrophages [22]. The M1 to M2 transition move to a critical role in inhibiting Achilles tendon adhesions and improving Achilles tendon strength by injecting cyclooxygenases (COX-1 and COX-2)-engineered miRNA plasmid/nanoparticles-loaded hydrogel into the injured Achilles tendon [50]. In this study, we studied the effects of composite materials on the regulation of immune microenvironment in vitro and in vivo. In the experimental results of macrophage induction on culture medium of TDSCs–SIS scaffold and TDSCs composite scaffold, it was found that the anti-inflammatory effect of the former was significantly stronger than the latter. RAW264.7, an accepted rat macrophage model, has been used to good effect in this experiment and in previous reports [51,52], but we still hope to confirm this finding by repeating the culture medium of TDSCs–SIS scaffold and TDSCs using primary cells. Meanwhile, the expression of CD163 was the highest in TDSCs–SIS group and showed a time-increasing pattern after the repair of Achilles tendon defect with this scaffold complex.

## 5. Conclusions

SIS scaffolds could promote the adhesion, proliferation, and tendonogenic differentiation of tendon stem cells. In the Achilles tendon defect model, implantation of TDSCs–SIS scaffold promotes regeneration of the Achilles tendon. Therefore, this study proposes a valuable biological scaffold to enhance the tendon repair ability of TDSCs. The resulting SIS scaffold and TDSCs synergistically promoted Achilles tendon regeneration, and M2 macrophage polarization was involved. The limitation of this study is that the in vivo transplantation of TDSCs was not labeled and further followed up.

## Figures and Tables

**Table 1:** Reverse transcription–polymerase chain reaction primer sequences.

Genes	Forward Primer Sequences	Reverse Primer Sequences
β-Actin	AGATGTGGATCAGCAAGCAG	GCGCAAGTTAGGTTTTGTCA
SCX	GACCCGCTTTCTTCCACAGC	GTCACGGTCTTTGCTCAACTTT
TNMD	GTGATTTGGGTTCCCGCAGAA	GTGGGATTGATCCAGTACATGG
COL1A1	ACGTCCTGGTGAAGTTGGTC	CAGGGAAGCCTCTTTCTCCT
COL3A1	CTGTAACATGGAAACTGGGGAAA	CCATAGCTGAACTGAAAACCACC
ARG-1	GGCTTGCTTCGGAACTCAAC	CATGTGGCGCATTCACAGTC
TGF-β	CCACCTGCAAGACCATCGAC	CTGGCGAGCCTTAGTTTGGAC
TNF-α	AGGCACTCCCCCAAAAGATG	TTGCTACGACGTGGGCTAC
iNOS	GTTCTCAGCCCAACAATACAAGA	GTGGACGGGTCGATGTCAC

Primer sequences were given in 5′ to 3′ direction.

## Data Availability

Data are contained within the article.

## Supplementary Materials

The following supporting information can be downloaded at: https://www.mdpi.com/article/10.3390/cells11172770/s1, Table S1: Histological scoring system; Figure S1: Masson tricolor staining of the coronal sections of the regenerated defects at 1, 2 and 3 months. Newly formed collagen fibers were visualized as blue and newly formed myofibrils were visualized as red. From loose and disordered collagen fibers to dense, orderly rows of collagen fibers, showing a time dependency, *n* = 4.
