# Supplementary material for: Enhancement of Tendon Repair Using Tendon-Derived Stem Cells in Small Intestinal Submucosa via M2 Macrophage Polarization
Source: Cells. 2022 Sep 5;11(17):2770. doi: 10.3390/cells11172770 (PMC9454771; doi:10.3390/cells11172770)
Supplement: Supplementary file 1 [file cells-11-02770-s001.zip › cells-1791668-supplementary.pdf]

Supplementary Table S1 Histological scoring system

	pt		pt
Extracellular matrix (ECM) organization of the whole tendon		*Cellularity/cell-matrix-ratio*	
		Physiological	2
Wavy, compact and parallel arranged collagen	2	Locally increased cell density	1
fibres	1	Increased cell density or decreased ECM	0
In part compact, in part loose or not orderly	0	content	
Loosely composed, not orderly ("granulation" tissue)			
Proteoglycan content (alcian blue staining)		*Cell distribution*	
Normal	1	Homogeneous, physiological	1
Focally increased	0	Focal areas of elevated cell density (cell clustering)	0
Cell alignment		*Transition from defect to normal tissue*	
Uniaxial	2	Scaffold integrated, no gaps at the	2
Areas of irregularly arranged cells (10e50%)	1	margin visible	
More than 50% of cells with no uniaxial	0	Recognizable transition	1
alignment		Abrupt transition, splitting/gaps detectable, callus tissue	0
Cell nucleus morphology		*Organization of repair tissue of the tendon*	
Predominantly elongated, heterochromatic cell	2	*callus*	
nuclei (tenocytes)		Homogeneous (whole tissue with similar	2
10-30% of the cells possess large, oval,	1	composition)	
euchromatic or polymorph heterochromatic		Locally heterogeneous tissue	1
nuclei	0	composition	0
Predominantly larger, oval, euchromatic or polymorph, heterochromatic nuclei		Whole tissue composition completely changed	
Configuration of callus		*Vascularisation in the defect area*	
Normal, only in the defect area, locally confined	1	Hypo-vascularized, like surrounding	1
Strong, change of whole tendon, thickened	0	tendon (small capillaries)	0
		Hyper-vascularized (increased numbers of small or larger capillaries)	
Degenerative changes/tissue metaplasia		*Inflammation*	
Non existing	3	No inflammatory cell infiltrates	1
Moderate formation of oedema	2	Infiltrating inflammatory cell types	0
Intense oedema with inclusion of fat, cell and/or fibres destruction, fibrin deposition, gaps	1	(neutrophils, macrophages, foreign-body/giant cell)	
Assembly of cartilage or bone (alcian blue-, von Kossa staining)	0		

pt; points.

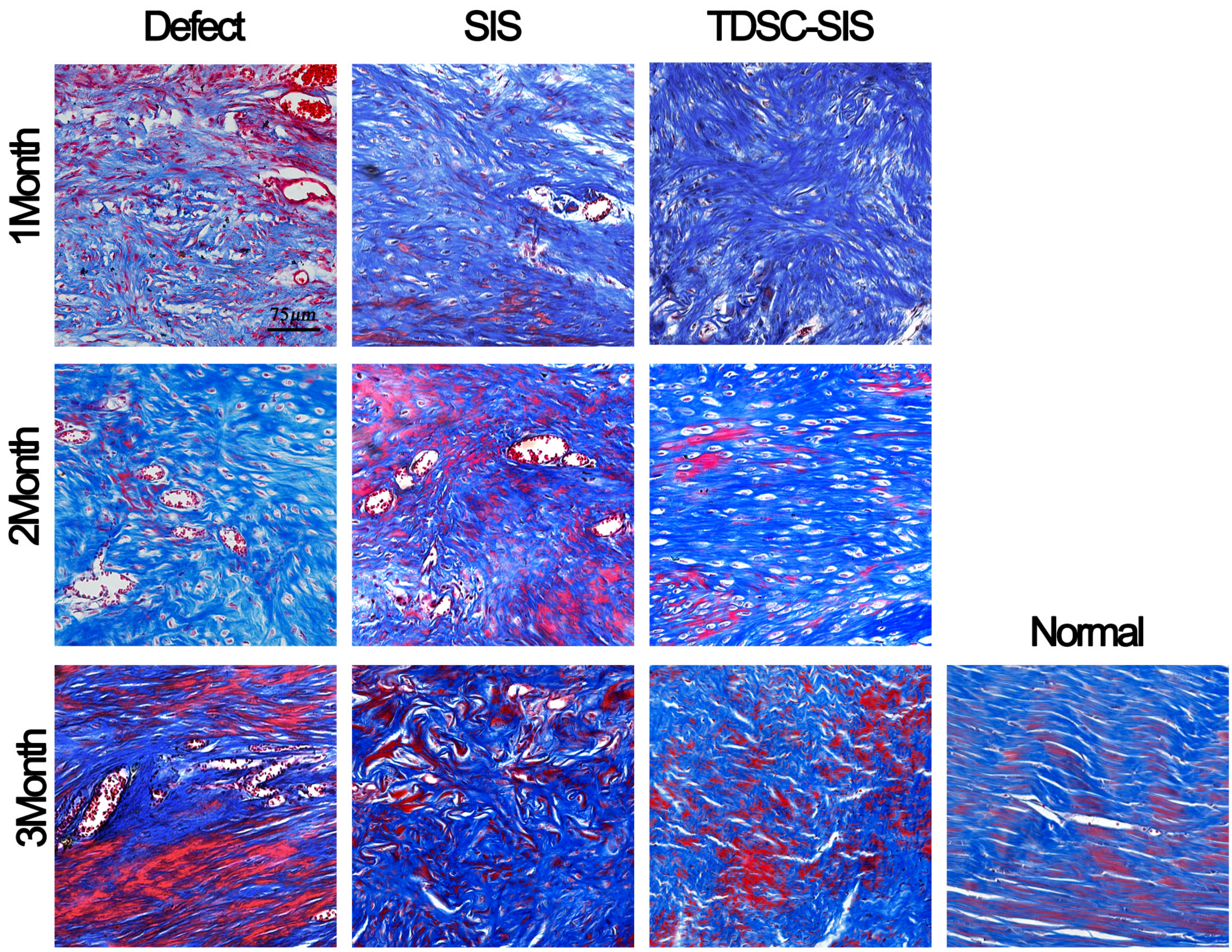

Supplementary Figure S1. Masson tricolour staining of the coronal-sections of the regenerated defects at 1，2 and 3 months. Newly formed Collagen fibres were visualized as blue and newly formed Myofibrils were visualized as red. From loose and disordered collagen fibres to dense, orderly rows of collagen fibres, showing a time dependency, n=4.
